# *Camellia neriifolia* and *Camellia ilicifolia* (Theaceae) as separate species: evidence from morphology, anatomy, palynology, molecular systematics

**DOI:** 10.1186/s40529-024-00430-2

**Published:** 2024-07-23

**Authors:** Zhaohui Ran, Zhi Li, Xu Xiao, Ming Tang

**Affiliations:** 1 College of Forestry, Guizhou University, Guiyang, 550025 China; 2 College of Forestry, Jiangxi Agricultural University, Nanchang, 330045 China; 3 Jiangxi Provincial Key Laboratory of Conservation Biology, Jiangxi Agricultural University, Nanchang, 330045 China; 4 Lushan National Observation and Research Station of Chinese Forest Ecosystem, Jiujiang, 332000 Jiangxi China

**Keywords:** *Camellia neriifolia*, *Camellia ilicifolia*, Molecular systematics, Separate species, Taxonomic revision

## Abstract

**Background:**

The systematic status of sect. *Tuberculata* and its taxonomy have recently attracted considerable attention. However, the different bases for defining the characteristics of sect. *Tuberculata* has led to many disagreements among the plants in this group. *Camellia neriifolia* and *Camellia ilicifolia* have been the subject of taxonomic controversy and have been treated as different species or varieties of the same species. Therefore, it is important to use multiple methods, i.e., integrative taxonomy, to determine the taxonomic status of *C. neriifoli*a and *C. ilicifolia*. This is the first study to systematically explore the taxonomic position of these two plants on the basis of Morphology, Anatomy, Palynology and Molecular Systematics.

**Results:**

Extensive specimen reviews and field surveys showed that many differences exist in *C. neriifolia* and *C. ilicifolia*, such as the number of trunk (heavily debarked vs. slightly peeling), leaf type (smooth thin leathery, shiny vs. smooth leathery, obscure or slightly shiny), leaf margin (entire vs. serrate), flower type (subsessile vs. sessile), number of styles (3–4 vs. 3), and sepal (ovate vs. round). Moreover, *C. neriifolia* has a more distinctive faint yellow flower color, and trunk molting was more severe in *C. neriifolia* than that in *C. ilicifolia*. In addition, micromorphological analysis of the leaf epidermis showed that the two species differed in the anticlinal wall, stomatal apparatus, and stomatal cluster, and pollen morphology analyses based on pollen size, germination furrow, and polar and equatorial axes showed that they are both distinct from each other. The results of the phylogenetic tree constructed based on the whole chloroplast genome, protein-coding genes, and ITS2 showed that both *C. ilicifolia* and *C. neriifolia* were clustered in different branches and gained high support.

**Conclusions:**

The results combine morphology, anatomy, palynology, and molecular systematics to treat both *C. neriifolia* and *C. ilicifolia* as separate species in the sect. *Tuberculata*, and the species names continue to be used as they were previously. In conclusion, clarifying the taxonomic status of *C. neriifolia* and *C. ilicifolia* deepens our understanding of the systematic classification of sect. *Tuberculata*.

**Supplementary Information:**

The online version contains supplementary material available at 10.1186/s40529-024-00430-2.

## Background

*Camellia* L. sect. *Tuberculate* H. T. Chang, which is set up on the basis of "*Camellia tuberculata* Chien", a species with "tuberculate protuberances on the surface of the fruits," is considered to be a specialized group of *Camellia* that retains its original shape (Chang 1981; Min and Zhong 1993). The classification of sect. *Tuberculate* began in 1939, and this section was set up by the famous botanist Prof. Chong-Shu Qian (Chien 1939). Subsequently, many new species within the tea group were gradually discovered (Chang 1981; Min and Zhong 1993; Chang and Ren 1991, 1996; Min 1999). Initially, Sealy divided the genus *Camellia* into 12 sections based on macroscopic morphological studies and field investigations, and *C. tuberculata* was placed under sect. *Heterogenea* Sealy (Sealy 1958). However, Chang (1981) thought that Sealy classification system does not reveal the evolutionary level or relationships of the divided taxa, thus he revised the genus *Camellia* and first proposed the section ‘*Tuberculate*’ on the basis of tuberculate shape of the fruit, and meanwhile, he named the newly discovered *C. ilicifolia* in Guizhou and assigned it to sect. *Pseudocamellia* Sealy Rev. *Camellia neriifolia*, another species in sect. *Tuberculate* described by him in 1984 (Chang 1984), is distinct by its glabrous shoots, cuneate or slightly rounded leaf base, glabrous capsules, verrucose protuberances, and other features in the protologue. It is noteworthy that the type locality of both species is Jinshagou, Chishui County, Guizhou Province. Min et al. revised the genus *Camellia* based on specimen studies, grouping the original 230 species into 119 taxa. He believed that there was an intimate evolutionary relationship between sect. *Pseudocamellia* and sect. *Tuberculate* and moved *C. ilicifolia* from sect. *Pseudocamellia* into sect. *Tuberculate*, and merged the *C. ilicifolia* with the *C. neriifolia* in a combined treatment (Min and Zhong 1993). However, Chang thought that the taxonomic traits of the two capsules show essential differences between *C. neriifolia* and *C. ilicifolia*, therefore he did not agree with Min and Zhong (Chang and Ren 1996). However, the taxonomic positions of these two species have not been accurately determined.

In recent years, Jiang (2010) classified *Camellia* L. based on leaf characteristics, and the findings indicated that the epidermal characteristics of the leaves of *C. neriifolia* and *C. ilicifolia* are similar, supporting the merger of the two. However, molecular phylogenetic of 146 species of plants within *Camellia* conducted by him (Jiang 2017) based on molecular data (*mat*K, *rbc*L, *ycf*1, and *trn*L-F) revealed that *C. neriifolia* and *C. ilicifolia* belong to different groups. Wu (2022) studied the phylogeny and evolution of secondary product metabolism in 166 species of *Camellia* L. using comparative transcriptomics, showed that sect. *Pseudocamellia* and sect. *Tuberculate* converges on the same branch. However, *C. ilicifolia* was not included in this study. In additon, the taxonomic status of *C. neriifolia* and *C. ilicifolia* remains unclear.

During plant evolution, the basic organizational structure and morphological features of plants have remained relatively stable and vary between species because of their genetics and adaptation to the environment (Zhou 2000). The flower, fruit, and leaf characteristics of plants have been widely used to classify Theaceae (Shen et al. 2008; Li et al. 2001; Luo et al. 1999). Pollen is a type of reproductive cell produced during plant reproduction (Hornick et al. 2021), and it has unique morphological features that can reflect the genetic and ecological characteristics of plants (Zhang et al. 2016). In plant classification, pollen features such as morphology, size, and outer wall ornamentation can be used to aid in identification (Fuchs 1967). Pollen ornamentation is diverse in type, complex in outer edge structure, and varies from one type of plant to another (Shi et al. 2022). Its morphology is mainly controlled by genes and is highly genetically conserved, providing a great deal of taxonomic information (Zhang et al. 2022). Previous studies have emphasized the importance of pollen morphology in Theaceae identification. Although sect. *Tuberculate* is a special group in Theaceae, there were few studies on pollen morphology and only sporadic reports on individual species (Wei et al. 1992). At present, there is no comprehensive field practice survey for *C. neriifolia* and *C. ilicifolia*, and their extant specimens are incomplete in terms of flower, fruit, and leaf characteristics. It is difficult to classify them on the basis of specimens, and they remain to be investigated. Chloroplast genome with relatively stable genetic information and high-resolution taxonomic information, thus revealing the evolution, origin, and affinity among species (Huang et al. 2022; Jiang et al. 2022). Therefore, the combination of morphological and phylogenetic analyses can provide more complete and accurate classification results (Lee and Palci 2015). In this study, *C. neriifolia* and *C. ilicifolia* were comparatively analyzed using traditional morphology, anatomy, palynology, and molecular systematics, to clarify the identities of the two taxa.

## Material and methods

### Materials collection

*C. neriifolia* and *C. ilicifolia* used in this study were obtained from Chishui City, Guizhou Province (former from Hutou Mountain, Yuanhou, and latter from Jinshagou). Photographs were obtained during the field surveys. The young leaves were preserved in sealed bags and subjected to DNA extraction. The specimens were maintained in the Tree Herbarium of the School of Forestry, Guizhou University (GZAC) (LZ-20221016, LZ-20221108).

### Morphology of plants

The habitats, trunks, branches, leaves, flowers, and fruits of both species were observed in their natural states using morphological methods. Leaf texture, color (both abxial and adaxial of the leaf), shape and shape of the leaf margin, leaf base, and leaf vein characteristics; petal size, number, color, presence or absence of persistent sepals, and number of styles; fruit shape, size, color, and folds of both species; and color and shape of the outer bark of the trunk and branches were observed. Statistical analyses were performed using Excel 2010 (Table S1).

### Plant leaf epidermis

Leaves from both plants (used for morphological observations) were cleaned and preserved by immersion in an FAA solution (70% ethyl alcohol, acetic acid, and formaldehyde in the ratio 90:5:5). When leaf observation was required, the leaves were removed from the FAA solution, and the residual solution was washed away with water. Leaf pieces were cut in the midvein region of the leaf according to an area of 0.5 cm × 0.5 cm, placed in 100% sodium hypochlorite, dissociated in a 30 °C thermostatic water bath, and then fished out when the leaf pieces were white. Leaf pieces were removed with tweezers and small razor blades to remove all leaf flesh and obtain a clean leaf epidermis. The clean upper and lower leaf epidermis was fashioned into clinical slices, stained with magenta acetate solution, placed under an optical microscope for careful observation, and the results obtained were kept in the image. The micromorphology of the upper and lower leaf epidermis, including the pericyclic wall and stomatal apparatus, was observed at different magnifications, and data were obtained and analyzed using image processing software.

The characteristics of stomata were from the fresh leaves of both species. Use a sharp blade to cut and harvest fresh tissue blocks quickly within 1–3 min. Leaf tissue was gently washed with PBS. The washed tissue blocks are immediately fixed by electron microscopy fixative for 2 h at room temperature, then transferred into 4 ℃ for preservation and transportation. Wash tissue blocks with 0.1 M PB (pH 7.4) for 3 times, 15 min each. Then transfer tissue blocks into 1% OsO4 in 0.1 M PB (pH 7.4) for 1–2 h at room temperature. After that, wash tissue blocks in 0.1 M PB (pH 7.4) for 3 times, 15 min each. Dry samples with Critical Point Dryer. Specimens are attached to metallic stubs using carbon stickers and sputter-coated with gold for 30 s. Observe and take images with scanning electron microscope.

### Microscopic morphology of pollen

For *C. neriifolia* and *C. ilicifolia*, respectively, the pollen of five healthy and consistent degrees of openness of the plant was collected according to the acetic anhydride decomposition method to deal with the pollen (Ao and Liu 2001), and then the treated pollen was placed under the scanning electron microscope, which can be observed and measured to determine the shape of pollen grains, the size of pollen grains, pollen germination grooves, pollen grains outer surface ornamentation, the equatorial plane view, and the polar surface view, and at the same time, leave the image to be preserved. The pollen images from the SEM were processed using the Image Tool image processing software to obtain data for the two species. The pollen size was expressed as the average of the equatorial (E) and polar (P) axes, and the length of the pollen germination furrow was measured at the same time.

### Chloroplast DNA extraction, assembly and annotation

The collected dried young leaves were used, and the modified CTAB method (Chen et al. 2004) was selected for the extraction of DNA from *C. neriifolia* and *C. ilicifolia* leaves. DNA integrity was detected by 1% agarose gel electrophoresis, and DNA concentration and purity were determined using a NanoDrop 2000 spectrophotometer. DNA was then subjected to random interruption, end repair, and junction ligation to construct sequencing libraries. Finally, the libraries that passed the quality test were sequenced using an Illumina high-throughput sequencing platform. Low-quality data were trimmed using the Trimmomatic software (Bolger et al. 2014). The filtered qualified CP sequences were compared with the sequences from sect. *Tuberculate* in the National Center for Biotechnology Information (NCBI) database and the filtered data were clipped from scratch using SOAPdenovo and NOVOPlasty software (Luo et al. 2012; Dierckxsens et al. 2017), to obtain the circular chloroplast genome sequence. Finally, the complete CP genome was obtained by online annotation, BLAST comparison, and manual correction, using *C. rubituberculata* Chang & Yu (MZ424202) as the reference sequence. The cp genome was mapped using the OrganellarGenomeDRAW (OGDRAW) software online tool (Lohse et al. 2007). ITS universal primers ITS1 and ITS4 (Xu et al. 2024), and PCR conditions were as follows: first 95 ℃ for 5 min, 1 cycle of 94 ℃ for 1 min, second 50 ℃ for 1 min, 72 ℃ for 50 s, 30 cycles of 95 ℃ for 1 min, then 50 ℃ for 1 min, 72 ℃ for 1 min, and 72 ℃ for 10 min. 72 ℃ for 1 min and 72 ℃ for 10 min. Finally, gel electrophoresis was performed, and the qualified samples were sent to Wuhan Prime Biotech for sequencing. The annotated chloroplast genome and ITS were uploaded to the NCBI database to obtain GenBank accession numbers.

### IR boundary expansion and contraction

The chloroplast genome sequences of *C. neriifolia* and *C. ilicifolia* were selected, and four chloroplast genome sequences were obtained from four sections. *Tuberculate* species data were downloaded from the NCBI for Biotechnology Information database. IR region boundary expansion and contraction analyses were performed, and comparative maps were plotted using IRscope (https://irscope.shinyapps.io/irapp/) (Amyiryousefi et al. 2018) online software.

### Phylogenetic tree analysis

The chloroplast genome sequences of 18 species of the genus *Camellia* were downloaded from NCBI, and 21 chloroplast genomes were phylogenetically analyzed using the species *Apterosperma oblata* (GenBank accession number: NC035641) as an outgroup. Sequence comparisons were performed using MAFFT 7 (Katoh and Standley 2014) phylogenetic trees were reconstructed using the maximum likelihood (ML) method in IQ-TREE v1.6.12 (Nguyen et al. 2015), manually corrected with Mega X (Kumar et al. 2018), and the best tree building model was selected. The optimal model (GTR + I + G) was determined using MrModeltest v2.3, and finally, a Bayesian (BI) phylogenetic tree was reconstructed using MrBayes v3.2.7 (Huelsenbeck and Ronquist 2001). Phylogenetic trees were constructed based on protein-coding genes with ITS2 in the same way as for the whole chloroplast genome. Finally, a phylogenetic tree was constructed using iTOL (Interactive Tree Of Life) v4 (https://itol.embl.de/) (Letunic and Bork 2021) online tool.

## Result

### Morphological characteristics of *C. neriifolia* and *C. ilicifolia*

**Fig. 1:**
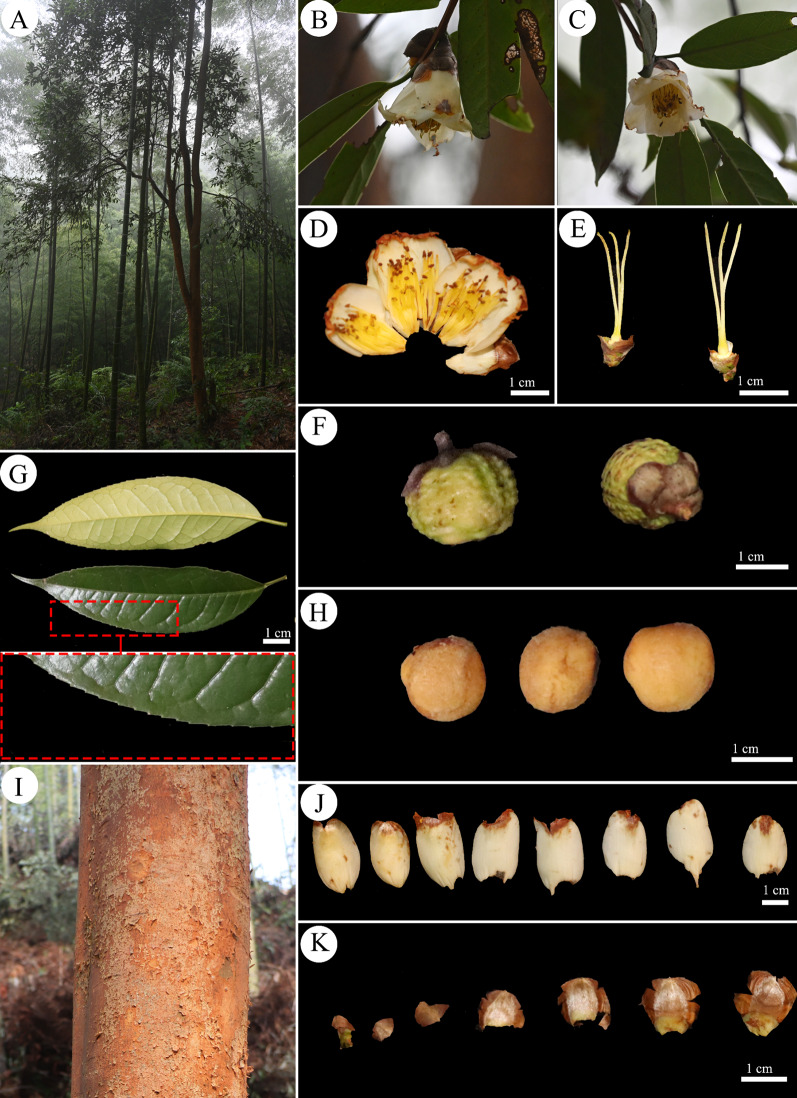
Morphological characters of *C. ilicifolia* (**A** Habitat; **B** and **C** Flowers; **D** Flower anatomy; **E** Style; **F** Fruit; **G** Leaves; **H** Seeds; **I** Trunk; **J** Petals; **K** Calyces.)

**Fig. 2:**
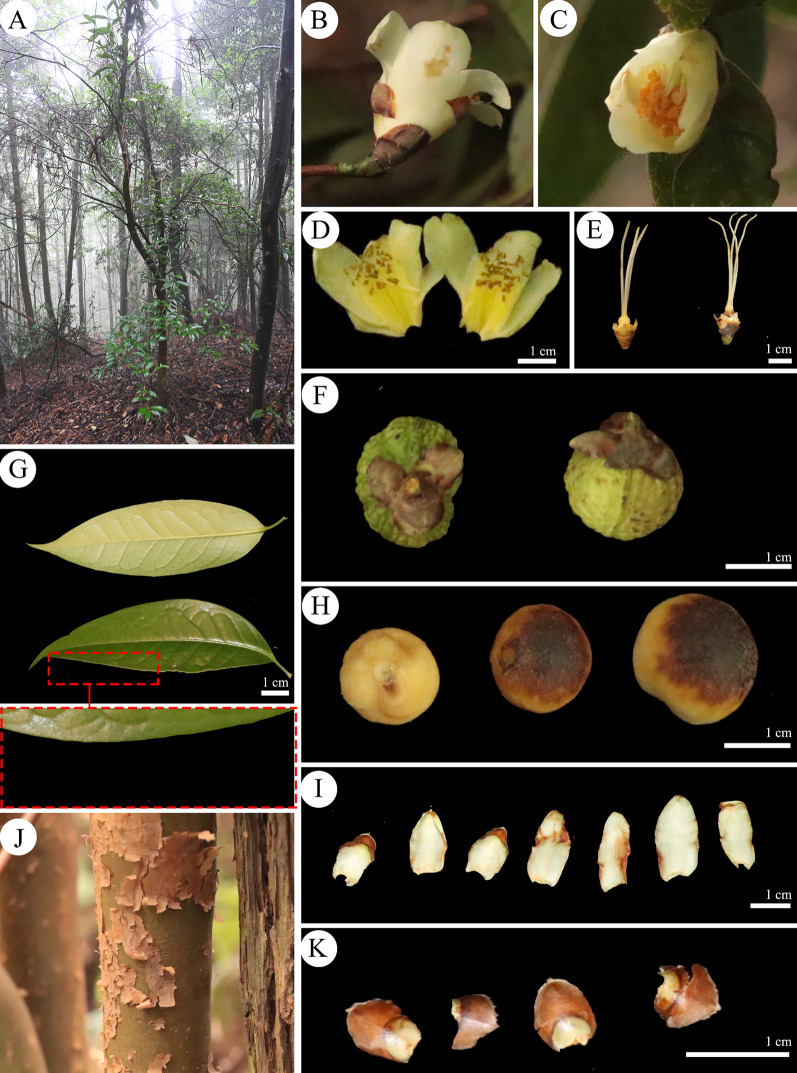
Morphological characters of *C. neriifoli*a (**A** Habitat; **B** and **C** Flowers; **D** Flower anatomy; **E** Style; **F** Fruit; **G** Leaves; **H** Seeds; **I** Trunk; **J** Petals; **K** Calyces.)

A detailed morphological comparison between the two species was performed. The results showed (Figs. 1, 2, Table 1) that there were some differences between the two species. For example, the differences in trunk (heavily debarked vs. slightly peeling), leaf type (smooth thin leathery, shiny vs. smooth leathery, obscure or slightly shiny), leaf margin (entire vs. serrate), flower type (subsessile vs. sessile), number of styles (3–4 vs. 3), and sepal (ovate vs. round) were more obvious between the *C. neriifolia* and *C. ilicifolia*.

**Table 1:** Comparison of morphological characters of *C. neriifolia* and *C. ilicifolia*

Species name	*C. neriifolia*	*C. ilicifolia*
Trunk	Reddish-brown, heavily debarked	Reddish brown, slightly peeling
Branches	Annual small-branched quadrangular, reddish brown	Annual small-branched quadrangular, reddish brown
Leaf type	Smooth thin leathery, shiny, narrowly lanceolate	Smooth leathery, obscure or slightly shiny, long oval or lanceolate
Leaf length (cm)	7.50–10.00	11.00–14.00
Leaf width (cm)	2.55–2.90	2.37–3.74
Leaf margin	Entire	Serrate
Petiole length (cm)	0.70–1.30	0.81–1.26
Flower type	Terminal, subsessile	Terminal, sessile
Flower colour	Faint yellow	White
Petal length (cm)	1.20–2.26	1.94–2.87
Petal width (cm)	0.80–1.20	1.30–1.80
Number of petals	6–8	8–12
Number of styles	3–4	3
Number of calyces	5–7	6–9
Sepal	Ovate	Round
Fruit shape	Capsule suborbicular, epidermis with verrucose projections	Capsule subglobose (lychee-like), epidermis with verrucose projections
Fruit diameter (mm)	14.0–19.8	13.0–18.5
Shell thickness (mm)	1.0–2.0	1.1–2.4
Ovaries	3–4	3–4
Habitat	Small trees, 1100–1200 m, under mixed montane and bamboo forests	Small trees or shrubs, forest edge, or bamboo forest around 950 m

### Leaf epidermal characteristics of *C. neriifolia* and *C. ilicifolia*

**Fig. 3:**
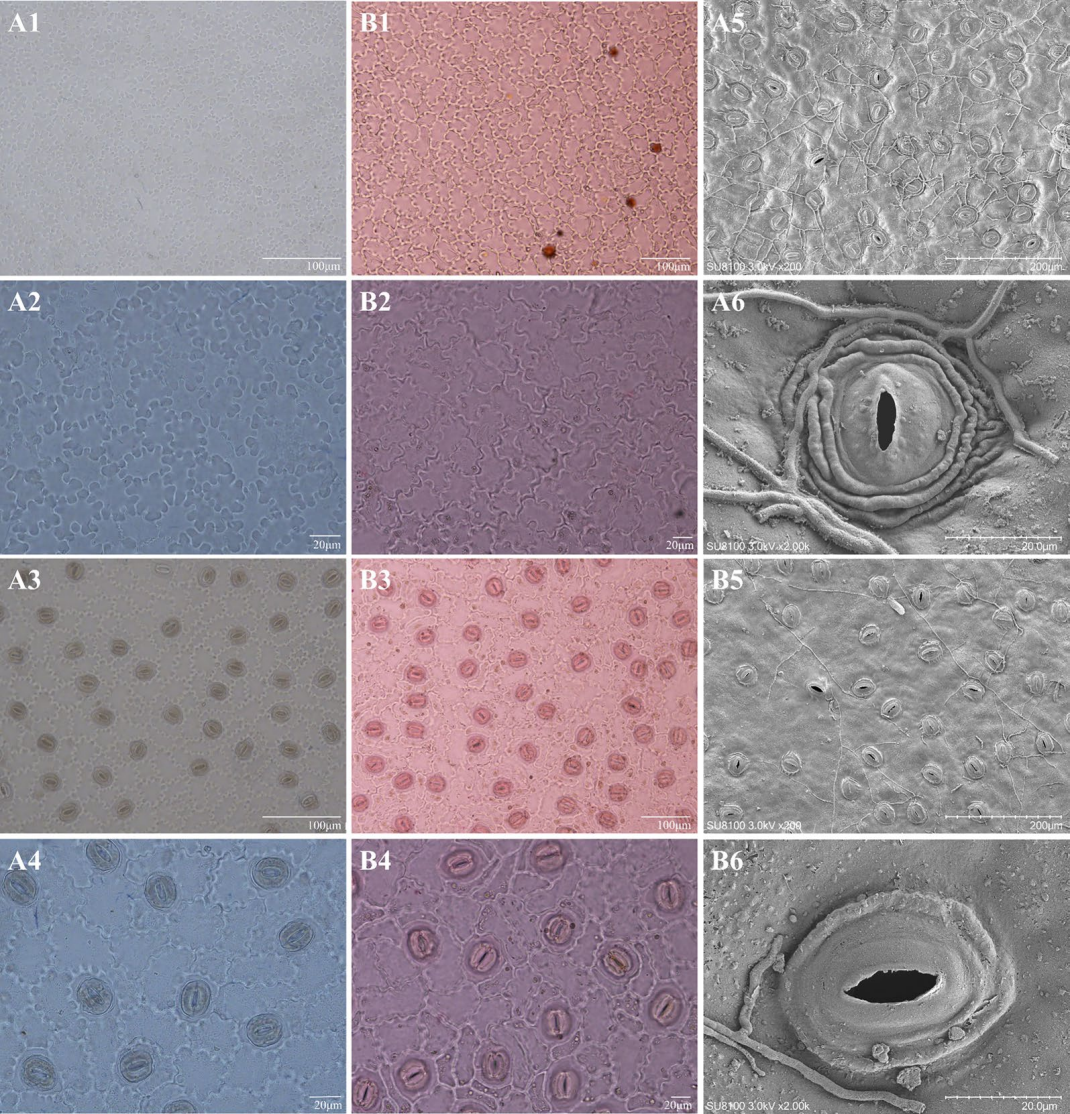
Leaf epidermal micromorphological characters (**A** *C. neriifolia*; **B** *C. ilicifolia* 1,2: Leaf upper epidermis; 3,4: leaf hypodermis; 1,3: 20 times; 2,4: 40 times; 5,6: Characters of stomata)

Images obtained under the electron microscope showed (Fig. 3, Table 2) that the epidermis of the *C. neriifolia* leaves be distinctly deeply wavy in the anticlinal wall of the upper epidermis and wavy in the lower epidermis. However, the leaf hypodermis of *C. ilicifolia* leaves was shallowly undulating, and that of the upper epidermis was shallower compared to that of the leaf hypodermis. The upper epidermis of the *C. neriifolia* has a smaller number of wavy peaks on the anticlinal wall, and the leaf hypodermis has more wavy peaks. In contract, the number of peaks in the periplane of the upper and leaf hypodermis of *C. ilicifolia* is less, and the leaf hypodermis is slightly more than that of the upper epidermis.

**Table 2:** Characteristics of leaf epidermis of *C. neriifolia* and *C. ilicifolia*

Species name	Pattern of anticlinal walls	Number of anticlinal wall crests	Sshape of stomata	Size of stomata (length/μm × width/μm)	Inner margin of outer rim	Cuticular ornamentation outside	Density of stomata (number/mm^2^)	Type of stomatal apparatus
*C. neriifolia*	The upper epidermis of the leaves is distinctly deeply undulate, and the leaf hypodermis is undulate	Leaf upper epidermis less numerous, leaf hypodermis more crested	Subrounded	18.00–25.00 × 19.00–24.00	Sinuolate	Ring edge, strip and ridge hunch	Occasional	Circular type
*C. ilicifolia*	Leaf hypodermis is shallowly undulate, the upper leaf epidermis is shallowly undulate slightly paler than leaf hypodermis	Leaf upper surface and leaf hypodermis skins less numerous, but leaf hypodermis slightly more numerous	Broadly elliptic	26.00–35.00 × 24.00–31.00	Sinuolate	Unconspicuous ring edge, a few ridge hunch	Unprecedented	Circular type

The stomatal apparatus (annular type, with three secondary guard cells surrounding the guard cells) of both *C. neriifolia* and *C. ilicifolia* was irregularly arranged and scattered only in the leaf hypodermis of the leaf blade, with no distribution observed in the upper epidermis. The stomatal apparatus of *C. neriifolia* was subrounded and occasional stomatal clusters were observed under a microscope. Two to three rings of folds were observed when the individual stomatal apparatus was magnified; these rings were presumed to be piled-up keratin-like material. The length of the stomatal apparatus was counted to be 18–25 μm, the width of the stomatal apparatus was 19–24 μm, and the stomatal density was 94–113 number/mm^2^. The stomatal apparatus of *C. ilicifolia* was broadly elliptic; no stomatal clusters were found under the microscope; no wrinkles were seen under magnification; the length of the stomatal apparatus was 26–35 μm, the width of the stomatal apparatus was 24–31 μm, and the stomatal density was 70–85 number/mm^2^ (Table S2).

The leaf epidermis of both plants were observed under a scanning electron microscope. The results showed (Fig. 3, Table 2) that the morphology of the inner margin of outer rim was sinuolate in both *C. neriifolia* and *C. ilicifolia.* However, the cuticular ornamentation outside of the former is an ring edge, strip and ridge hunch, and that of the latter is an unconspicuous ring edge, a few ridge hunch.

### Palynology characteristics of *C. neriifolia* and *C. ilicifolia*

**Fig. 4:**
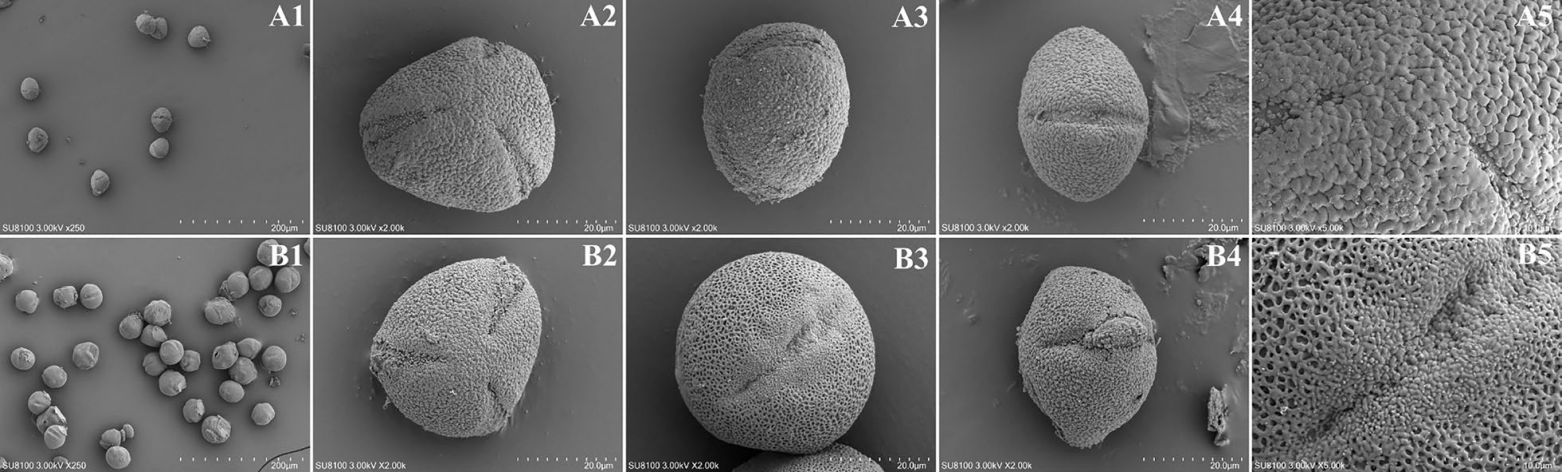
Electron microscope scanning pollen morphology characteristics of *C. neriifolia* and *C. ilicifolia*. **A** *C. neriifolia* and **B** *C. ilicifolia*: 1. Ensemble view; 2. Polar view; 3. Equatorial view; 4. germination grooves; 5. Pollen wall ornamentation

The results of the study showed (Table 3, Fig. 4) that the size of *C. ilicifolia* pollen grains was 31–35 μm, the morphology of pollen grains was oblate spherical, trilobate orthotropic in polar view, pike-shaped in equatorial view, the polar-anodal ratio was 0.73–0.75, P × E = 24.74–25.15 × 33.56–33.92 μm, and pollen germination pores were of 3-pore furrow type; the pollen wall ornamentation was wrinkled wave to granular, with coarse reticulation ridges, shallow inter-ridge stripes, and deep and wide reticulation mesh. The size of *C. neriifolia* pollen grains was 32–36 μm; the morphology of the pollen grains was subglobular, trilobate deltoid in polar view and ellipsoid in equatorial view; the polar to equatorial ratio was 1.03–1.05, P × E = 36.14–36.72 × 35.06–35.64 μm; the pollen germination pore was of 3-pore furrow type, with constriction in the middle of the furrow; the pollen wall ornamentation was crumpled wave to granular, with thicker and obvious ridges and warty grains; and the reticulum was obvious, with a deep and wide reticulum.

**Table 3:** Morphological characteristics of the pollen of *C. neriifolia* and *C ilicifolia*

Species	Shape of pollen	Polar axis (P)/μm	Equatorial axis (E)/μm	P/E	Germination pores/μm			Pollen wall ornamentation
					Long	Wide	L/W	
*C. ilicifolia*	Oblate	27.74–25.15	33.56–33.92	0.73–0.75	21.26–21.74	5.11–5.43	3.91–4.25	Conspicuous
*C. neriifolia*	Subglobular	36.14–36.72	35.06–35.64	1.03–1.05	28.04–28.47	5.17–5.33	5.26–5.51	Conspicuous

The pollen micromorphology of both species showed three germination furrows, but the length and type of the germination furrow varied. The length of the germination furrow of the *C. neriifolia* was about 28.04–28.47 μm; the germination furrow was deep and narrow, and the aperture furrow was long, thin, and narrow. The length of the *C. ilicifolia* germination pores is about 21.26–21.74 μm; the germination furrow is shallow, wide pike-type, and the aperture furrow gradually becomes wider and narrower from the two ends to the middle and stands out in the middle.

### Characterization of *C. neriifolia* and *C. ilicifolia* chloroplast genomes

**Fig. 5:**
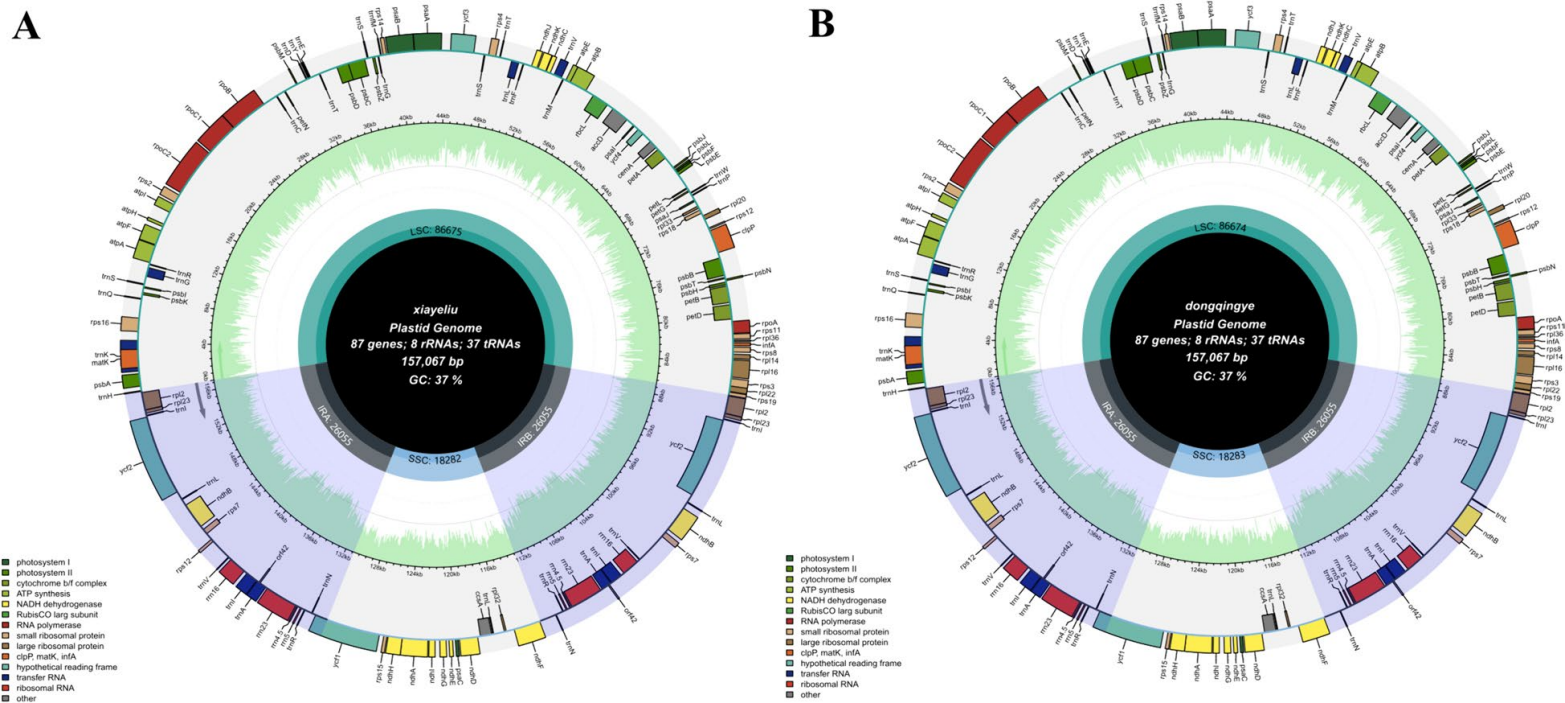
Genome mapping of *C. neriifolia* (**A**) and *C. ilicifolia* (**B**)

The whole chloroplast genomes of these two species were submitted to the NCBI GenBank, and GenBank accession numbers were obtained (Table 4). The cp genomes of both species were 157,067 bp in length (Table 4). Similar to most angiosperms, both have a typical tetrameric structure (Fig. 5). It comprises an LSC region, an SSC region, and two IR regions. The total number of genes in both species was 132, including 87 CDS, 37 tRNAs, and 8 rRNAs genes (Table 4). The total GC content of the entire cp genome was 37.3%. However, there were differences in the GC content of each region, with the IR region having a manifestly higher GC content than that of the LSC or SSC regions (Table 4).

**Table 4:** Genome-wide characterization of chloroplasts of *C. neriifolia* and *C. ilicifolia*

Species	C. *neriifolia*	*C. ilicifolia*
Genome size (bp)	157,067	157,067
GC (%)	37.3	37.3
LSC size (bp)	86,675	86,674
SSC size (bp)	18,282	18,283
IR size (bp)	52,110	52,110
GC in LSC (%)	35.31	35.31
GC in SSC (%)	30.61	30.61
GC in IR (%)	42.96	42.97
GC in CDS (%)	37.61	37.61
1st position GC (%)	45.37	45.37
2nd position GC (%)	38.04	38.04
3rd position GC (%)	29.46	29.43
Length of CDS	79,099	79,160
Number of genes	132	132
Number of CDS	87	87
Number of tRNA	37	37
Number of rRNA	8	8

**Fig. 6:**
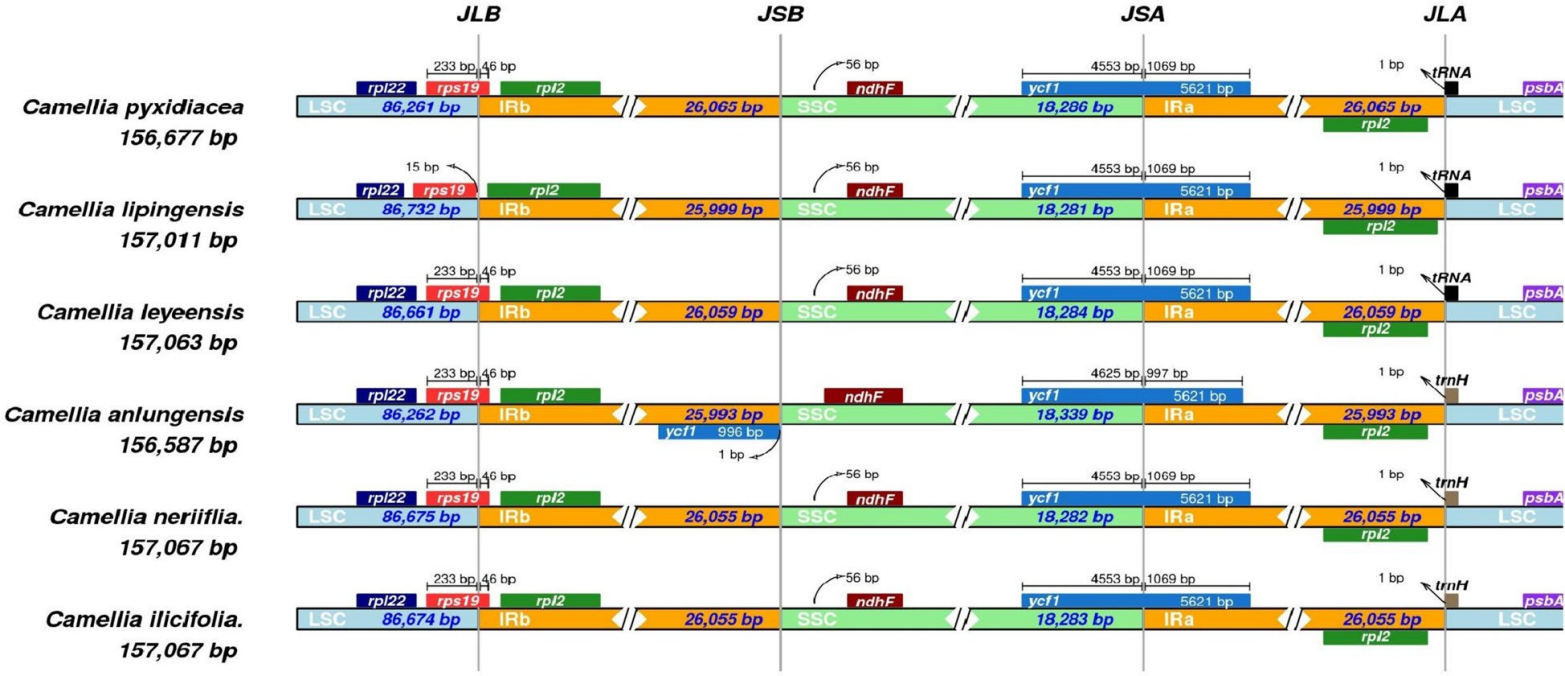
Analysis of contraction and expansion of the IR boundaries of the *C. neriifolia and C. ilicifolia*

The IR boundary contraction and expansion analysis (Fig. 6) showed that the *rps19*, *rpl2* and *trnH* genes were relatively conserved, with the same gene positions and lengths in *C. neriifolia* and *C. ilicifolia*. However, interspecific differences were not obvious, with only slight differences in the lengths of the LSC regions. However, these two plants were compared with *C. lipingensis*, *C. pyxidiacea*, *C. leyeensis* and *C. anlungensis. C. anlungensis*, *C. neriifolia* and *C. ilicifolia* were located on the trnH gene, and *C. lipingensis*, *C. pyxidiacea*, and *C. leyeensis* were located on tRNA genes. The tendency of the six plants to contract and expand the IR boundaries was basically the same.

### Phylogenetic tree analysis

**Fig. 7:**
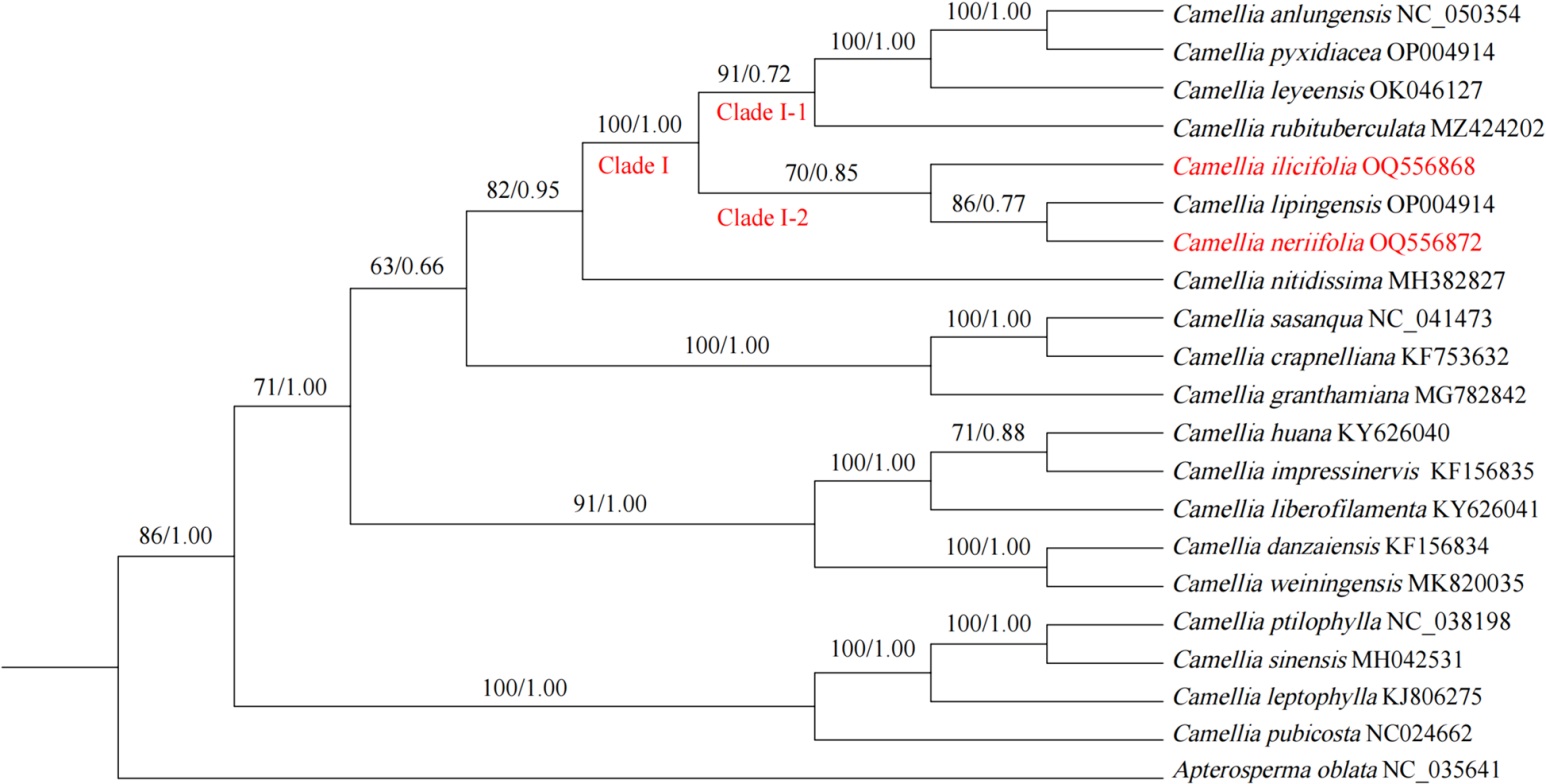
Construction of a phylogenetic tree based on the chloroplast genome (Maximum Likelihood (ML) and Bayesian (BI) trees, BS ≥ 50% and PP ≥ 0.95 are indicated above branches as BS/PP)

**Fig. 8:**
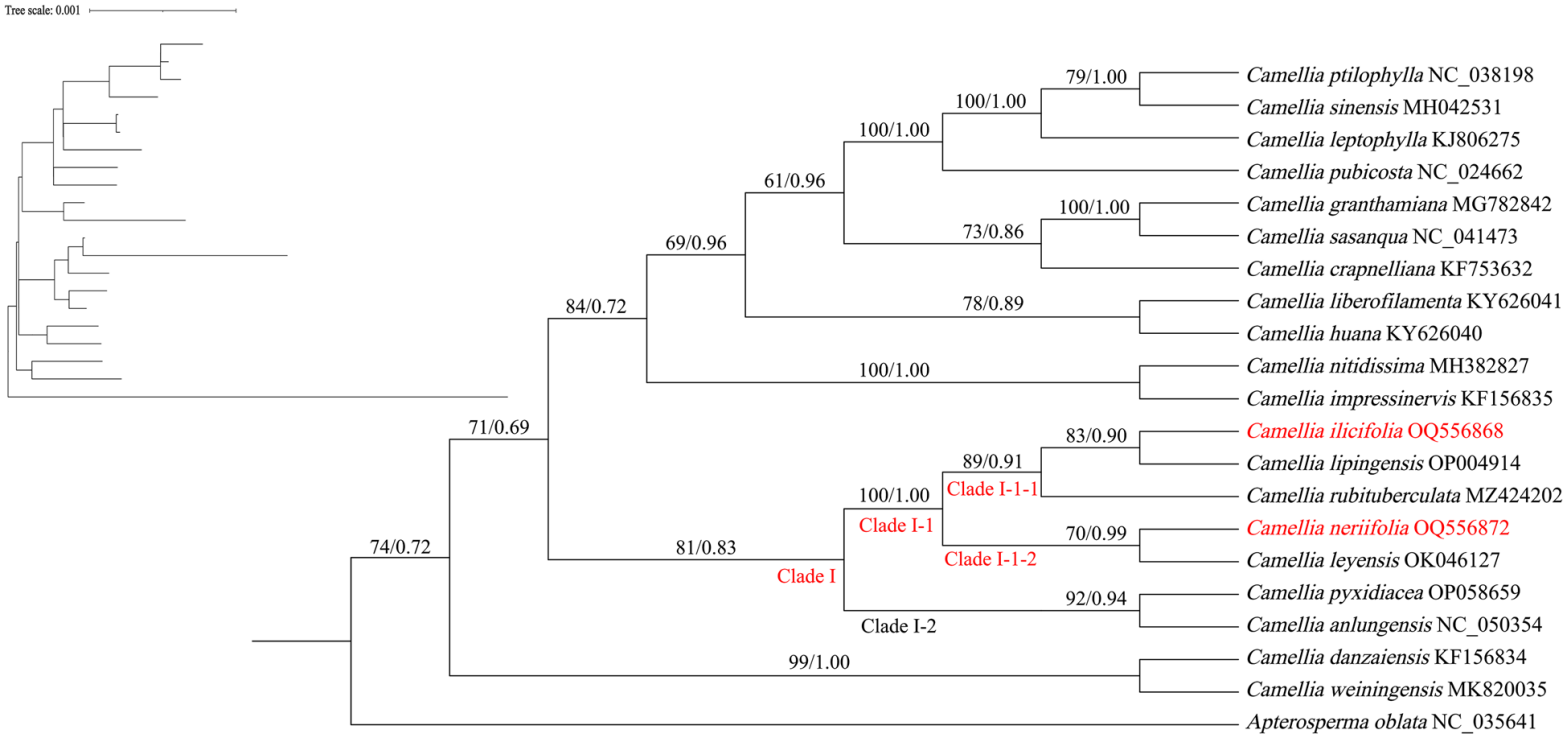
Construction of a phylogenetic tree based on the PCGs (Maximum Likelihood (ML) and Bayesian (BI) trees, BS ≥ 50% and PP ≥ 0.95 are indicated above branches as BS/PP)

**Fig. 9:**
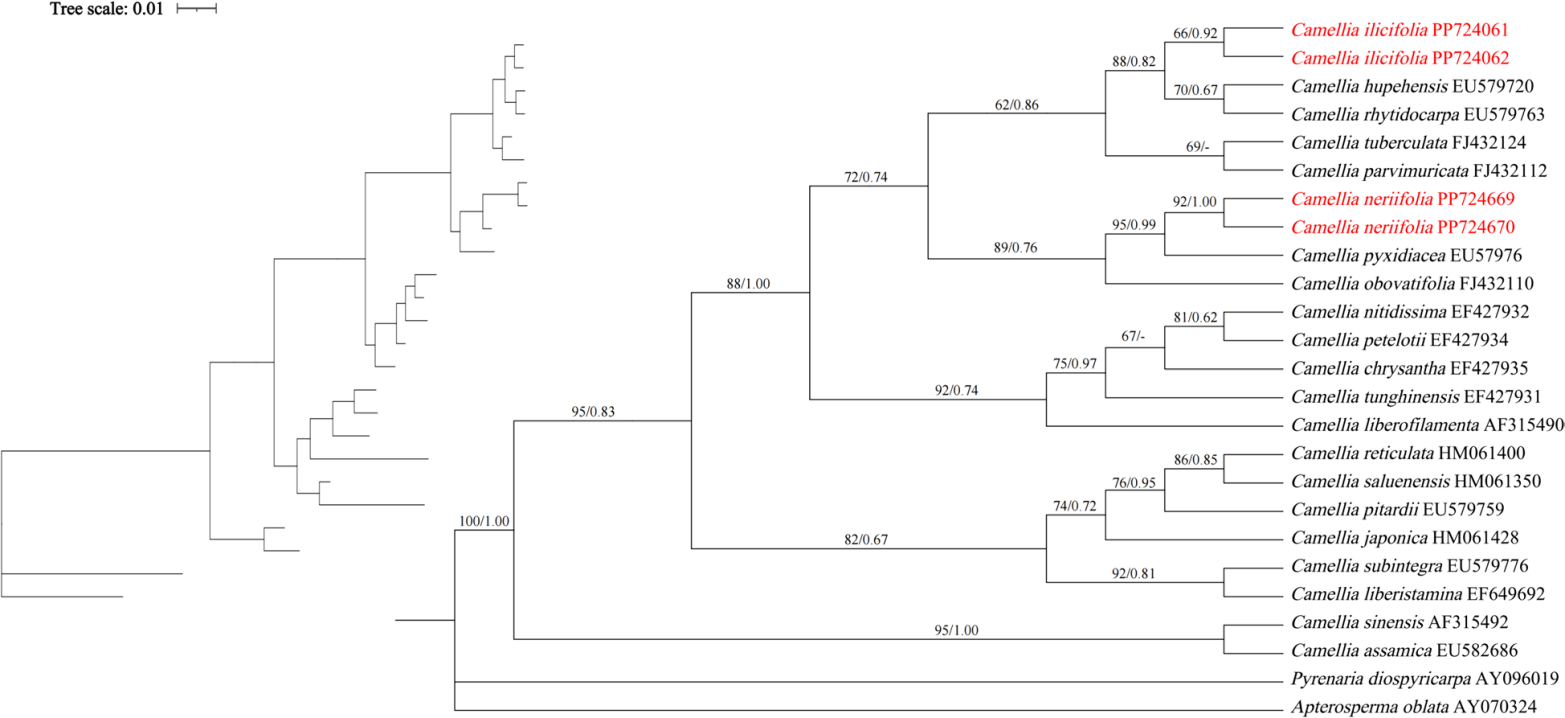
Construction of a phylogenetic tree based on the ITS2 (Maximum Likelihood (ML) and Bayesian (BI) trees, BS ≥ 50% and PP ≥ 0.95 are indicated above branches as BS/PP)

Based on the results of the chloroplast whole-genome phylogenetic tree of 21 species (Fig. 7), *C. ilicifolia* clustered with seven *C. tuberculata* in Clade I and had a high support rate (ML = 100, BI = 1.00). *C. neriifolia* and *C. ilicifolia* are on the same branch of Clade I-2 but not clustered together (ML = 70, BI = 0.85). Meanwhile, the results of the phylogenetic tree constructed on the basis of protein-coding genes and ITS2 sequences showed that both *C. neriifolia* and *C. ilicifolia* were clustered in the tea group, and the two species were clustered in different branches with high support (Figs. 8, 9).

### Taxonomic treatment

Combined the aforementioned evidence such as morphological, anatomy, and palynology fetures, as well as molecular phylogenetic results, it is demonstrated that *C. ilicifolia* and *C. neriifolia* act as independent species and both should be placed in sect. *Tuberculate*. Therefore, they are classified as two separate species:

#### *Camellia neriifolia* Chang

*Camellia neriifolia* Chang, in Acta Sci. Nat. Univ. Sunyat. (23)2: 79, 1984; *C. ilicifolia* var. *neriifolia* Ming, in Act. Bot. Yunania, 15(2): 125, 1993 et 21(2): 156, 1999.

**Fig. 10:**
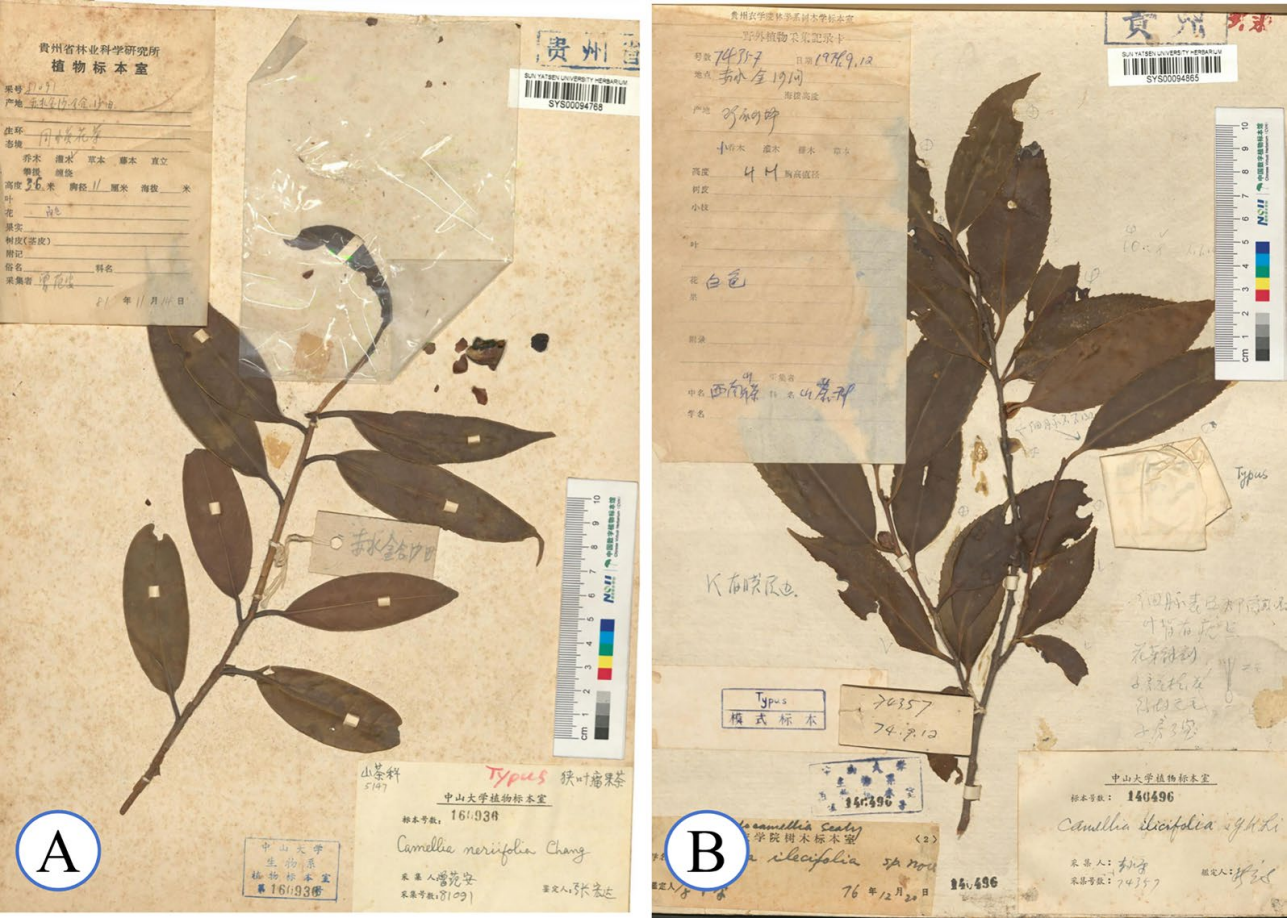
Information on type specimens of *C. neriifolia* and *C. ilicifolia* (**A** *C. neriifolia*, **B** *C. ilicifolia*)

**Type:** CHINA: Chishui, Jinsha, Shatian, Zeng Fanan 81,091 (SYS, GZFI) (Fig. 10A).

**Description.** Small macrophanerophytes, leaf blade thinly leathery, smooth and glabrous, dark green, obscure or slightly shiny adaxially, yellowish green abaxially; leaf blade symmetrical, narrowly lanceolate; length usually about 7.50–10.00 cm, width 2.55–2.90 cm; leaf apex caudate-acuminate, base broadly cuneate; leaf margins smooth, without any teeth or notches joining in a line; leaf parallel lateral veins 7–9 per side, visible above, more obvious below; reticulate veins not easily visible above, clearly visible below; petiole length 0.70–1.30 cm; flowers terminal, subsessile; flowers with 5–7 sepals, scarious, reddish-brown, 1.21–1.72 cm long, 0.44–0.81 cm wide, slightly ovate at the apex, similar to a fingernail cap, slightly hairy; petals 6–8 in number, faint yellow, 1.20–2.26 cm long, 0.80–1.20 cm wide; pistil with 3–4 styles, free, glabrous. Capsules are globose and glabrous, 14.0–19.8 mm in diameter, with a surface with verrucose projections. Fruit 3-loculed and 1-seeded per locule, seeds globose, approximately 0.68–1.64 cm in diameter, hairy. The fruiting pedicels are very short, with persistent sepals at the posterior end of the fruit. The branchlets are reddish-brown, and shoots are glabrous and glossy when dry. The trunk was yellowish-brown, with varying degrees of desiccation, and the trunk and branches were smooth with fine lines.

**Fig. 11:**
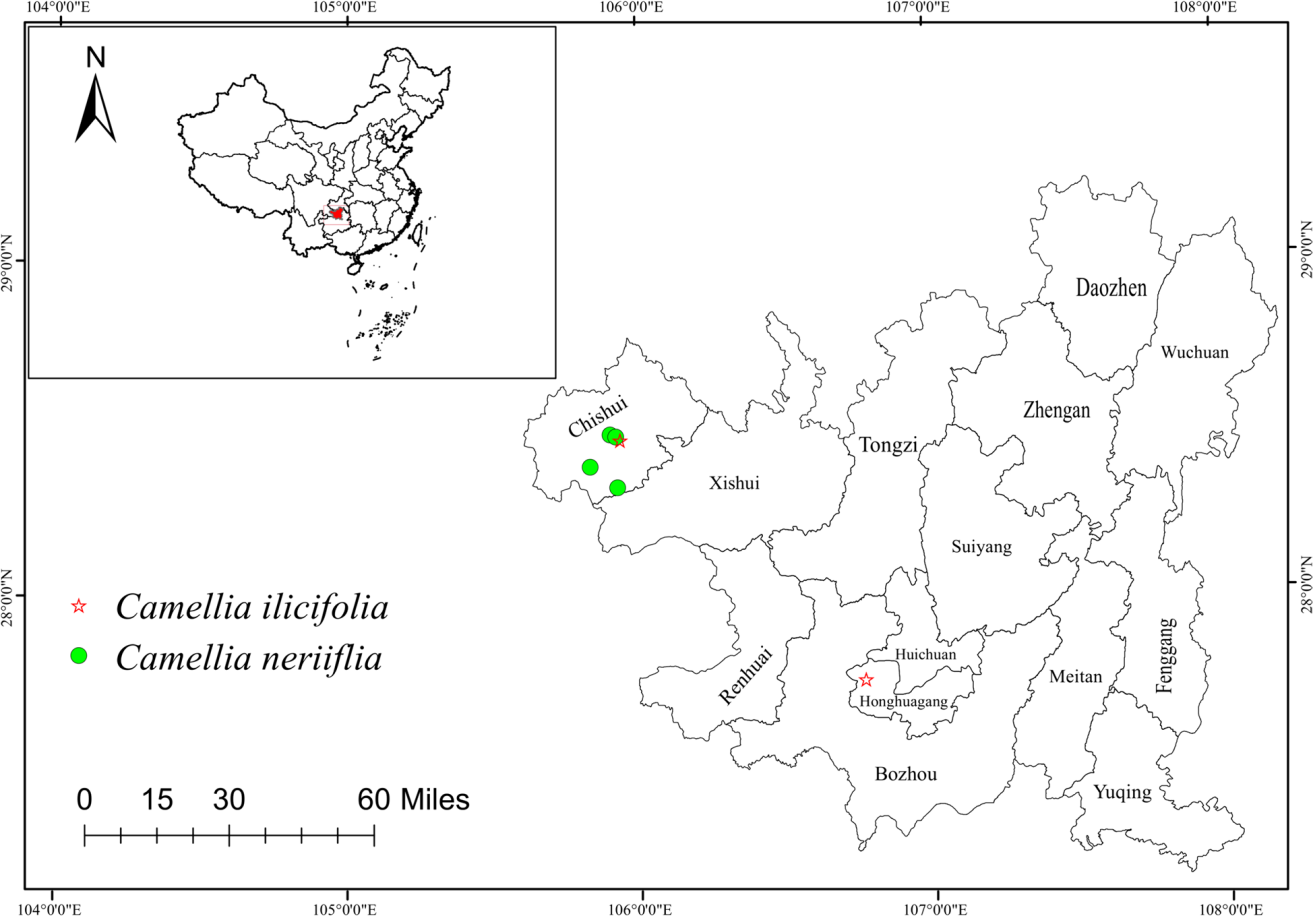
Distribution map of the species of *C. neriifolia and C. ilicifolia*

**Distribution and habitat:** Endemic to Guizhou, distributed in Chishui: Hutou Mountain in Yuanhou, Jinhe Village in Jinsha, Wild Boar Ping, and Stuffy Head Creek (Fig. 11). Most grow on steep slopes at 1100–1200 m above sea level, under mixed woods and bamboo forests in the mountains, sometimes forming small groups.

#### *Camellia ilicifolia* Y. K. Li

*Camellia ilicifolia* Y. K. Li & H. T. Chang, Tax. *Camellia* 46, 1981; H. T. Chang and B. Bartholomew, Camellia 66, 1984; Flora of Guizhou, 5:9, 1988.

**Type:** CHINA: Chishui, Li YongKang 74,357 (SYS, GZAC) (Fig. 10B).

**Description.** Shrubs or small macrophanerophytes, leaf blade leathery, smooth and glabrous, adaxially dark green, glossy, abaxially yellow-green; leaf blade symmetrical, long elliptic or lanceolate; leaf blade length usually around 11.00–14.00 cm, width 2.37–3.74 cm; leaf apex acuminate or caudate-acuminate, base cuneate or rounded; leaf blade margins uniformly acute serrulate with regular inter-tooth intervals; proximal serrations sparser than distal ones; leaf margins glandular; parallel lateral veins 7–9 per side, visible on both sides; reticulate veins visible on both sides, clearer below; petiole length about 0.81–1.26 cm; flowers terminal, sessile; flowers with 6–9 sepals, scarious, reddish-brown, 1.46–1.88 cm long, 0.81–1.13 cm wide; apex slightly rounded, similar to fingernail shape; slightly hairy. Petals number 8–12, white, 1.30–1.80 cm wide, 1.95–2.87 cm long; pistil with 3 styles, free, glabrous. Capsules are subglobose and glabrous, about 13.0–18.5 mm in diameter, with a surface with verrucose projections. Fruit 3-loculed and 1-seeded per locule, seeds orbicular, approximately 0.84–2.13 cm in diameter, hairy. The fruiting pedicels are very short, with persistent sepals at the posterior end of the fruit. The branchlets are reddish-brown, and shoots are glabrous and glossy when dry. The trunk was yellowish-brown, with varying degrees of desiccation, and the trunk and branches were smooth with fine lines.

**Distribution and habitat:** Endemic to Guizhou, distributed in Taojiapo, Jindingshan, Zunyi, Jinshagou, and Chishui (Fig. 11). Most plants grow in steep forests and valleys, usually at an altitude of approximately 950 m, sometimes form small colonies. It grows at an altitude of 950 m.

## Discussion

*C. neriifolia and C. ilicifolia* are controversial in traditional taxonomy in terms of ovary, leaves, and calyx (Min and Zhong 1993; Chang and Ren 1996). After reviewing a large number of specimens and field surveys, we found that leaf shape, petiole length, and fruit size were fairly stable among plant species populations, suggesting that these characteristics are key features of sect*. Tuberculata*. The leaves of seed plants evolved from a primitive branching system (Efroni et al. 2010). Fruit morphology is an important taxonomic tool (Song and Hong 2020). We found that fruit morphology was a very stable trait in both plants, making fruit difficult to use as key evidence. By observing the morphology, structure, characteristics, flowering period, and inflorescence of flowers, plants can be classified and identified, and their evolution and kinship can be studied (Decraene and Smets 1994). We found that these two species did not differ manifestly in calyx, flower type, or flower color. However, there were manifest differences between the two species in terms of trunks, leaf type, leaf margin, flower type, number of styles, and sepals. These characteristics provide an important taxonomic basis for the morphological delineation of the two species.

Leaf epidermal micromorphological features can be used as the basis for plant classification (Stace 1966; Brittan 1970; Kong 2001). In the taxonomic study of the genus *Camellia*, the difference in the number of upper and lower epidermal anticlinal wall crests can reflect interspecific differences to a certain extent, and the combination of the taxonomic indices of the anticlinal wall styles will be more conducive to the discovery of evolutionary relationships and developmental patterns between the two species. From these data, it is clear that the stomatal apparatus of *C. ilicifolia* is larger than that of *C. neriifolia*, and the species identity is obvious. This result is consistent with that of previous studies (Jiang 2010). Thus, the leaf epidermal microstructures of both plants provided manifest evidence for the establishment of the two separate species. According to Erdtman's criteria for classifying pollen size, the pollen grains of both plants were small to medium-sized (Erdtman 1969). Morphological and data analyses of the pollen showed manifest differences in pollen shape, germination pores, and polar axes, supporting the independence of the two species. For example, the pollen morphology of *C. neriifolia* is sub-globose, whereas that *C. ilicifolia* is oblate. This is consistent with previous results (Wei et al. 1992; Hu et al. 2021).

Whole chloroplast genomes can provide powerful genetic resources for molecular phylogenetic studies of wild plant resources. Phylogenetic analyses have greatly deepened our understanding of the evolutionary relationships between *C. neriifolia* and *C. ilicifolia*. According to the molecular results of the present study, *C. neriifolia and C. ilicifolia* clustered on the same branch Clade I-2 with high support, which demonstrated the close affinity of the two species. Based on our fieldwork, Chishui Jinshagou was the most concentrated and dominant distribution area for both species. These sites highly overlap, and it is hypothesized that a high degree of natural hybridization may exist. The two species may have evolved over different periods.

## Conclusion

In this study, we analyzed and compared for the first time the morphology, anatomy, palynology and molecular systematics traits of *C. neriifolia* and *C. ilicifolia*. The results showed that although not all traits were of systematic and taxonomic significance, some of them played a key role in the differentiation of the species, such as leaf margin type and sepal shape. Leaf epidermis, palynology and phylogeny also provide evidence for the independence of the two species. Therefore, both *C. neriifolia* and *C. ilicifolia* were treated as separate species in the sect. *Tuberculata* in this study. It provides an important reference value for the genetic diversity assessment, phylogeny, and population genetics of this species.

### Supplementary Information

Supplementary material 1.

Supplementary material 2.

## Data Availability

GenBank accession numbers: OQ556872, OQ556868, PP724061-PP724062, and PP724669-PP724670. The Appendix Table for this article can be found online.

## References

[CR1] Amyiryousefi A, Hyvonen J, Poczai P (2018) IRscope: an online program to visualize the junction sites of chloroplast genomes. Bioinformatics 34(17):3030–3031. 10.1093/bioinformatics/bty220 29659705 10.1093/bioinformatics/bty220

[CR2] Ao CQ, Liu XK (2001) A simple method for preparing pollen specimen in light microscope. Chin Bull Bot 18(2):251

[CR3] Bolger AM, Lohse M, Usadel B (2014) Trimmomatic: a flflexible trimmer for illumina sequence data. Bioinformatics 30(15):2114–2120. 10.1093/bioinformatics/btu170 24695404 10.1093/bioinformatics/btu170 PMC4103590

[CR4] Brittan NH (1970) A preliminary survey of the stem and leaf anatomy of Thysanotus R. Br. (Liliaceae). Bot J Linnean Soc 3(1):57–70

[CR5] Chang HT (1981) Systematic study of the genus *Camellia*. In Journal of Sun Yatsen University (Natural Science Edition) Forum, pp 47–52

[CR6] Chang HT (1984) New record of *Camellia* from South China. Acta Scientiarum Naturaliun Universitatis Sun Yatseni 23(2):77–82

[CR7] Chang HT, Ren SX (1991) A classification on the section *Tuberculata* of *Camellia*. Acta Scientiarum Naturaliun Universitatis Sunyatseni 30(4):86–91

[CR8] Chang HT, Ren SX (1996) Diagnosis on the systematic development of *Camellia* VI. Revised on sect. *Tuberculata* of *Camellia*. Suppl J Sun Yatsen Univ 2:55–60

[CR9] Chen KS, Li F, Xu CJ, Zhang SL, Fu CX (2004) An efficient macro-method of genomic DNA isdation from *Actinidia chinensis* leaves. Hereditas 26(4):529–531 15640056

[CR10] Chien SS (1939) Four new ligneous plants from Szechuan. Contr Biol Lab Sci Soc China Bot Ser 12(2):89–100

[CR11] Decraene LR, Smets EF (1994) Merosity in flowers: definition, origin, and taxonomic significance. Plant Syst Evol 191:83–104. 10.1007/BF00985344 10.1007/BF00985344

[CR12] Dierckxsens N, Mardulyn P, Smits G (2017) NOVOPlasty: de novo assembly of organelle genomes from whole genome data. Nucleic Acids Res 45(4):e18. 10.1093/nar/gkw955 28204566 10.1093/nar/gkw955 PMC5389512

[CR13] Efroni I, Eshed Y, Lifschitz E (2010) Morphogenesis of simple and compound leaves: a critical review. Plant Cell 22(4):1019–1032. 10.1105/tpc.109.073601 20435903 10.1105/tpc.109.073601 PMC2879760

[CR14] Erdtman G (1969) Handbook of palynology, an introduction to the study of pollen grains and spores. Munksgaard, Copenhagen

[CR15] Fuchs HP (1967) Pollen morphology of the family Bombacaceae. Rev Palaeobot Palynol 3:119–132. 10.1016/0034-6667(67)90045-0 10.1016/0034-6667(67)90045-0

[CR16] Hornick T, Richter A, Harpole WS, Bastl M, Bohlmann S, Bonn A et al (2021) An integrative environmental pollen diversity assessment and its importance for the sustainable development goals. Plants People Planet 4(2):110–121. 10.1002/ppp3.10234 10.1002/ppp3.10234

[CR17] Hu ZM, Zhao YY, Zhao CH, Liu JX (2021) Pollen morphology of Liliaceae and its systematic significance. Palynology 45:531–568. 10.1080/01916122.2021.1882601 10.1080/01916122.2021.1882601

[CR18] Huang YY, Li J, Yang ZR, An WL, Xie CZ, Liu SS, Zheng XS (2022) Comprehensive analysis of complete chloroplast genome and phylogenetic aspects of ten *Ficus* species. BMC Plant Biol 22:253. 10.1186/s12870-022-03643-4 35606691 10.1186/s12870-022-03643-4 PMC9125854

[CR19] Huelsenbeck JP, Ronquist F (2001) MRBAYES: Bayesian inference of phylogenetic trees. Bioinformatics 17(8):754–755. 10.1093/bioinformatics/17.8.754 11524383 10.1093/bioinformatics/17.8.754

[CR20] Jiang ZD (2017) Preliminary study of molecular phylogenetics and biogeography of the genus *Camellia* L. based on chloroplast DNA. Dissertation, Zhejiang Sci-Tech University

[CR21] Jiang B, Peng QF, Shen ZG, Moller M, Pi EX, Lu HF (2010) Taxonomic treatments of *Camelliaa* (Theaceae) species with secretory structures based on integrated leaf characters. Plant Syst Evol 290:1–20. 10.1007/s00606-010-0342-x 10.1007/s00606-010-0342-x

[CR22] Jiang H, Tian J, Yang JX, Dong X, Zhong ZX, Mwachala G et al (2022) Comparative and phylogenetic analyses of six Kenya *Polystachya* (Orchidaceae) species based on the complete chloroplast genome sequences. BMC Plant Biol 22:177. 10.1186/s12870-022-03529-5 35387599 10.1186/s12870-022-03529-5 PMC8985347

[CR23] Katoh K, Standley DM (2014) MAFFT: iterative refinement and additional methods. Methods Mol Biol 1079:131–146. 10.1007/978-1-62703-646-7_8 24170399 10.1007/978-1-62703-646-7_8

[CR24] Kong HZ (2001) Comparative morphology of leaf epidermis in the Chloranthaceae. Bot J Linn Soc 136(279–294):294. 10.1111/j.1095-8339.2001.tb00573.x 10.1111/j.1095-8339.2001.tb00573.x

[CR25] Kumar S, Stecher G, Li M, Knyaz C, Tamura K (2018) MEGA X: molecular evolutionary genetics analysis across computing platforms. Mol Biol Evol 35(6):1547–1549. 10.1093/molbev/msy096 29722887 10.1093/molbev/msy096 PMC5967553

[CR26] Lee MY, Palci A (2015) Morphological phylogenetics in the genomic age. Curr Biol 25(19):R922–R929. 10.1016/j.cub.2015.07.009 26439355 10.1016/j.cub.2015.07.009

[CR27] Letunic I, Bork P (2021) Interactive Tree Of Life (iTOL) v5: an online tool for phylogenetic tree display and annotation. Nucleic Acids Res 49(W1):293–296. 10.1093/nar/gkab301 10.1093/nar/gkab301 PMC8265157 33885785

[CR28] Li FY, Wang YG, Tang SQ (2001) Characters of leaf epidermis in section chrysantha series chrysantha (Theaceae, *Camellia*) and their systematic significance. J GuangXi Norm Univ 4:75–79

[CR29] Lohse M, Drechsel O, Bock R (2007) Organellar Genome DRAW (OGDRAW): a tool for the easy generation of highquality custom graphical maps of plastid and mitochondrial genome. Curr Genet 52(5):267–274. 10.1007/s00294-007-0161-y 17957369 10.1007/s00294-007-0161-y

[CR30] Luo CQ, Tan XF, Qi LL (1999) A classification summary on plant of Genus *Camellia*. J Cent South For Univ 19(03):78–81

[CR31] Luo RB, Liu BH, Xie YL, Li ZY, Huang WH, Yuan JY et al (2012) SOAPdenovo2: an empirically improved memory-effificient short-read de novo assembler. Gigascience 1:18. 10.1186/2047-217X-1-18 23587118 10.1186/2047-217X-1-18 PMC3626529

[CR32] Min TL (1999) A systematic synopsis of the genus *Camellia*. Acta Bot Yunnanica 21(2):149–159

[CR33] Min TL, Zhong YC (1993) A revision of genus *Camellia* Sect. Tuberculata Acta Botanica Yunnanica 15(2):123–130

[CR34] Nguyen LT, Schmidt HA, Haeseler A, Minh BQ (2015) IQTREE: a fast and effective stochastic algorithm for estimating maximum-likelihood phylogenies. Mol Biol Evol 32(1):268–274. 10.1093/molbev/msu300 25371430 10.1093/molbev/msu300 PMC4271533

[CR35] Sealy JR (1958) A revision of the genus *Camellia*. The Royal Horticulture Society, London, pp 1–239

[CR36] Shen JB, Lv HF, Peng QF, Zheng JF, Tian YM (2008) FTIR spectra of *Camellia* sect. *Oleifera*, sect. *Paracamellia*, and sect. *Camellia* (Theaceae) with reference to their taxonomic significance. J Syst Evol 46(2):194–204. 10.3724/SP.J.1002.2008.07125 10.3724/SP.J.1002.2008.07125

[CR37] Shi SY, Wu WF, Cui J, Zhang YY, Li ZH, Wang Y (2022) Pollen morphology and taxonomic significance of ten species of sect. Chrysantha Guihaia 42(1):68–77

[CR38] Song HJ, Hong SP (2020) Fruit and seed micromorphology and its systematic significance in tribe *Sorbarieae* (Rosaceae). Plant Syst Evol 306:6. 10.1007/s00606-020-01640-4 10.1007/s00606-020-01640-4

[CR39] Stace CA (1966) The use of epidermal characters in phylogenetic considerations. New Phytol 65:304–318. 10.1111/j.1469-8137.1966.tb06366.x 10.1111/j.1469-8137.1966.tb06366.x

[CR40] Wei ZX, Zavada MS, Min TL (1992) Pollen morphology of *Camellia* (Theaceae) and ITS taxonomic significance. Acta Bot Yunnanica 14(3):275–282

[CR41] Wu Q, Tong W, Zhao HJ, Ge RH, Li RP, Huang J, Li FD et al (2022) Comparative transcriptomic analysis unveils the deep phylogeny and secondary metabolite evolution of 116 *Camellia* plants. Plant J 111:406–421. 10.1111/tpj.15799 35510493 10.1111/tpj.15799

[CR42] Xu Q, Yang L, An MT, Yu JH, Liu F, Li Z (2024) *Ixeridium malingheense* (Asteraceae), a new species from southwestern Guizhou, China. Phytotaxa 645(20):163–171. 10.11646/phytotaxa.645.2.5 10.11646/phytotaxa.645.2.5

[CR43] Zhang DB, Shi JX, Yang XJ (2016) Role of lipid metabolism in plant pollen exine development. Subcell Biochem 86:315–337. 10.1007/978-3-319-25979-6_13 27023241 10.1007/978-3-319-25979-6_13

[CR44] Zhang JL, Lin JB, Li TF, Liu HP (2022) Research advances in pollen morphology of *Camellia*. Southeast Hortic 10(6):470–475. 10.20023/j.cnki.2095-5774.2022.06.015 10.20023/j.cnki.2095-5774.2022.06.015

[CR45] Zhou YB (2000) Plant morphological anatomy experiment. Beijing Normal University Press, Beijing

